# Supplementary material for: Resolving the full spectrum of human genome variation using Linked-Reads
Source: Genome Res. 2019 Apr;29(4):635–45. doi: 10.1101/gr.234443.118 (PMC6442396; doi:10.1101/gr.234443.118)
Supplement: Supplemental Material [file supp_gr.234443.118_Supplemental_longranger-2.2.zip › longranger-2.2.tar.bz2!/lr2.2-public/lib/stan/cmdstan/stan/doc/api/html/structstan_1_1gm_1_1integrate__ode.html]

Stan: stan::gm::integrate\_ode Struct Reference


|  |  |
| --- | --- |
|  | Stan  2.5.0  probability, sampling & optimization |


- Main Page
- Related Pages
- Namespaces
- Classes
- Files

- Class List
- Class Index
- Class Hierarchy
- Class Members

All Classes Namespaces Files Functions Variables Typedefs Enumerations Enumerator Friends Macros Pages

- stan
- gm
- integrate\_ode

Public Member Functions |
Public Attributes |
List of all members

stan::gm::integrate\_ode Struct Reference

`#include <ast.hpp>`

|  |  |
| --- | --- |
| Public Member Functions | |
|  | integrate\_ode () |
|  | |
|  | integrate\_ode (const std::string &system\_function\_name, const expression &y0, const expression &t0, const expression &ts, const expression &theta, const expression &x, const expression &x\_int) |
|  | |

|  |  |
| --- | --- |
| Public Attributes | |
| std::string | system\_function\_name\_ |
|  | |
| expression | y0\_ |
|  | |
| expression | t0\_ |
|  | |
| expression | ts\_ |
|  | |
| expression | theta\_ |
|  | |
| expression | x\_ |
|  | |
| expression | x\_int\_ |
|  | |

## Detailed Description

Definition at line 332 of file ast.hpp.

## Constructor & Destructor Documentation

|  |  |  |  |  |
| --- | --- | --- | --- | --- |
| stan::gm::integrate\_ode::integrate\_ode | ( |  | ) |  |

Definition at line 835 of file ast\_def.cpp.

|  |  |  |  |
| --- | --- | --- | --- |
| stan::gm::integrate\_ode::integrate\_ode | ( | const std::string & | *system\_function\_name*, |
|  |  | const expression & | *y0*, |
|  |  | const expression & | *t0*, |
|  |  | const expression & | *ts*, |
|  |  | const expression & | *theta*, |
|  |  | const expression & | *x*, |
|  |  | const expression & | *x\_int* |
|  | ) |  |  |

Definition at line 836 of file ast\_def.cpp.

## Member Data Documentation

std::string stan::gm::integrate\_ode::system\_function\_name\_

Definition at line 333 of file ast.hpp.

expression stan::gm::integrate\_ode::t0\_

Definition at line 335 of file ast.hpp.

expression stan::gm::integrate\_ode::theta\_

Definition at line 337 of file ast.hpp.

expression stan::gm::integrate\_ode::ts\_

Definition at line 336 of file ast.hpp.

expression stan::gm::integrate\_ode::x\_

Definition at line 338 of file ast.hpp.

expression stan::gm::integrate\_ode::x\_int\_

Definition at line 339 of file ast.hpp.

expression stan::gm::integrate\_ode::y0\_

Definition at line 334 of file ast.hpp.

---

The documentation for this struct was generated from the following files:

- src/stan/gm/ast.hpp
- src/stan/gm/ast\_def.cpp


---

[ Stan Home Page ]

*© 2011–2014,
Stan Development Team.*
